# Household and School-Level Influences on Smoking Behavior among Korean Adolescents: A Multilevel Analysis

**DOI:** 10.1371/journal.pone.0098683

**Published:** 2014-06-04

**Authors:** Jongho Heo, Juhwan Oh, S. V. Subramanian, Ichiro Kawachi

**Affiliations:** 1 Public Health Joint Doctoral Program, San Diego State University & University of California San Diego, La Jolla, California, United States of America; 2 JW LEE Center for Global Medicine, Seoul National University College of Medicine, Seoul, Korea; 3 Department of Social and Behavioral Science, Harvard School of Public Health, Boston, Massachusetts, United States of America; University College London, United Kingdom

## Abstract

**Background:**

Trends in adolescent smoking rates in South Korea have not shown substantial progress due to a lack of effective anti-smoking interventions and policies in school settings.

**Methods and Findings:**

We examined individual- and school-level determinants of adolescent smoking behavior (ever smoking, current smoking, and daily smoking) using the nationally representative fifth Korean Youth Risk Behavior Web-based Survey conducted in 2009. We found that students in coeducation schools or vocational high schools had greater risks of smoking for each type of smoking behavior than those in single-sex schools or general high schools, respectively even after controlling for individual-level factors. Higher family affluence and higher weekly allowances were associated with greater risks of ever smoking, current smoking and daily smoking even after controlling for parental education and other confounders.

**Conclusions:**

Whilst caution is required in interpreting results given the cross-sectional nature of the study, our findings suggest that in addition to raising the price of cigarettes, youth anti-smoking interventions in South Korea may benefit from focusing on coeducation schools and vocational high schools.

## Introduction

Curbing smoking among adolescents has proved remarkably challenging in spite of strenuous efforts in high-income and middle-income countries [1]. Some middle-income as well as most low-income countries still lack the political will and resources to implement comprehensive anti-smoking policies to reverse the trends in youth smoking [2]. South Korea also has suffered from a lack of policies such as strong school-based anti-smoking interventions, increasing taxation on tobacco products, or bans on advertising and sponsorship from tobacco companies, even in the aftermath of the passage of the National Health Promotion Act (1995) and the Youth Protection Law (1997) [3]. Consequently, adolescent smoking rates in Korea persist near the top of Organization for Economic Cooperation and Development (OECD) countries (about 16.8% adolescents are current smokers in Korea) [4].

To give implications for anti-smoking interventions, researchers have conceptualized smoking behavior as a developmental continuum [5]–[7]. Although there are variations in definitions and terminology across studies, five states are recognized: pre-contemplation, contemplation/preparation, initiation/experimentation, intermittent/sporadic smoking, and daily/established smoking [8], [9]. Importantly, there may be different determinants of the different states of the smoking continuum, and previous studies have sought to identify the triggers of progression between different states. Of the individual risk factors, being male [6] and white [10], having a positive attitude toward smoking [11], and frequent alcohol and other drug use [12] were related to experimentation and progression to regular smoking. Lower educational achievement [13], [14] and depressive symptoms [15], [16] were uniquely related to progression to regular and daily smoking. Given that adolescents are susceptible to external and peer influences, past studies on adolescent smoking also have highlighted that close social bonding is one of the major risk factors related to smoking onset and transition to higher stages of smoking intensity. Having more smokers among close friends [6], [17], [18] was also a strong predictor of smoking initiation and regular smoking. Family influences such as having more smokers among family members [6], [19] and permissive parental attitudes regarding children’s smoking [10] were additional major contributors to adolescent regular smoking.

Despite the accumulated evidence on social environmental influences on adolescent smoking, there is still a scarcity of studies on the influence of parental socioeconomic status (SES) and school environment with the developmental continuum framework, particularly in the Asian context. The past studies that examined the relationship between parental SES and adolescent smoking showed mixed results. Studies have shown that higher levels of parental education have been associated with lower risk of adolescent smoking initiation [20], regular smoking [16], as well as higher rates of smoking cessation [21]. Household income and parental occupational status also showed inverse associations with adolescent current smoking (intermittent smoking and daily smoking) [22] and daily smoking [23]. However, other studies have failed to find a link between parental SES and adolescent smoking [24]–[27]. A few studies even reported associations in the opposite direction [28]: for example, in one study among high school students in US, higher parental income, occupational status, and education attainment were associated with increased risk of adolescent smoking [29].

Another inconsistent finding in smoking literature is the association between weekly allowances or “pocket money” for adolescents and risk of smoking. Chen et al. [30] and McNeill et al. [31] reported a positive association of adolescents’ disposable income with smoking initiation, experimental smoking, and regular smoking among UK and Chinese adolescents. Soteriades and DiFranza [16] reported that among US adolescents living in Massachusetts, higher weekly allowance was positively correlated with smoking even after controlling for parental education in the model (however, the association became statistically insignificant after controlling for household income). West et al. [32] found that both weekly allowance and family SES (measured by parental social class) was positively associated with smoking among Scottish adolescents; however, allowances had stronger effects on adolescent current and daily smoking for those from higher class backgrounds. Lastly, Do and Finkelstein [33] found no association between weekly allowance and smoking initiation among Korean adolescents. The inconsistencies in the literature may be due to differences in the inclusion of covariates in regression models. In examining the associations between parental SES and adolescent smoking, only a few previous studies controlled for a comprehensive set of confounders including depression [34], weekly allowance [35], and parental smoking [36].

Another environmental influence to be more examined on adolescent smoking is the school context. Recently, the influence of school contexts on adolescent smoking has begun to be examined via multilevel analytic methods identifying influential school-level characteristics related to adolescent smoking. Kandel et al. [37] found that a higher percentage of racial minorities in school was associated with decreased risk of daily smoking. Johnson and Hoffmann [38] found that a higher proportion of students from the same race/ethnic background in school significantly reduced daily smoking risk among black and Hispanic students. They also found that higher level of academic competition in the school increases the risk of daily smoking. Leatherdale et al. [39] showed higher smoking rates among seniors at school increased the odds of intermittent and regular smoking among Canadian adolescents. Consistent with a study among Canadian junior students [40], Murnaghan et al. [41] showed an association between smoking rates among seniors at school and increased risk of smoking initiation among US junior students. Similarly, higher smoking prevalence of a school was also associated with increased risk of students’ current smoking [42]. Schools with anti-smoking programs and clear policies and rules about non-smoking were protective against progression to intermittent smoking [41] and regular smoking [43], [44]. Lower teacher workload was also a protective factor against progression to regular smoking [43]. However, few multilevel studies have been conducted outside western countries to examine the influence of school contexts on adolescent smoking.

It is well known that the patterns of adolescent smoking are embedded in cultural contexts. In most Asian countries, under the Confucian culture that concerns gender-appropriate behavior, boys’ smoking is consistently more prevalent than among girls (albeit smoking rates are rising even among girls) [4], [45], [46]. Confucian culture is also reflected in the educational system, for example, public single-sex high schools are still favored in many Asian countries under the Confucian system, reflecting traditional gender roles and norms. Whether these Asian-specific school contexts influence smoking behaviors among Asian adolescents differently compared with Western school contexts remains an open question [47]–[49].

To fill these gaps, we first sought to estimate the associations of parental SES with adolescent smoking behaviors, carefully controlling for potential confounders including stress, depressive symptoms, parental smoking, and adolescent’s weekly allowance in our analytic models. Secondly, we sought to estimate potential associations of school-contextual factors with smoking states among Korean adolescents using nationally representative data with multilevel statistical framework, which enabled us to consider individual- and school-level factors simultaneously [50]. Lastly, we examined whether different factors are associated with different states of adolescent smoking.

## Methods

### Source of Data

We analyzed data from the Fifth Korean Youth Risk Behavior Web-based Survey (KYRBWS), a nationally representative repeated cross-sectional sample of 75,066 individuals nested in 800 schools. The ethics committee of the Korea Centers for Disease Control and Prevention (KCDC) approved the survey and the KCDC conducted the survey in 2009 and publicly released the data in 2010. The present analysis was exempt from institutional review board review as we used a de-identified publicly available secondary dataset. The KYRBWS used a stratified two-stage (schools and classes) cluster sampling approach to obtain a nationally representative sample. Based on the sampling design, KYRBWS sampled 76,937 adolescents (13–18 year olds) among middle schools (n = 400) and high schools (n = 400). Written informed consent was obtained from each student’s parents for the survey. Sampled students recorded their responses anonymously online during one hour of their regular class time. The response rate of the study was 97.6%. After excluding missing values for parental education variables, we identified 57,857 students from 400 middle and 400 high schools for the analysis.

### Measurement

**Figure 1:**
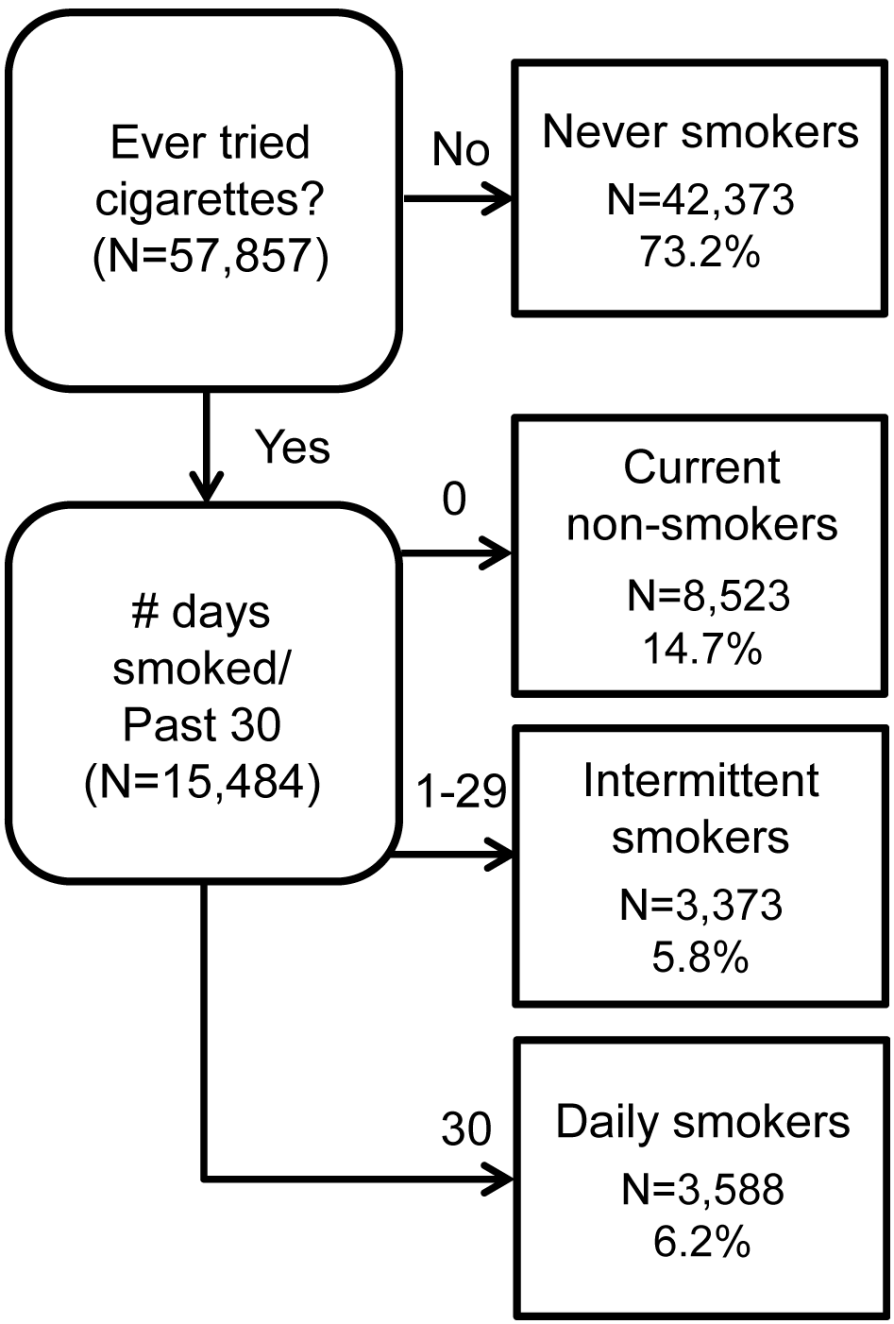
Branch diagram of adolescent smoking state and corresponding sample sizes.

We defined four types of smoking behaviors, which were drawn from the survey questionnaires: never smokers, current non-smokers, intermittent smokers, and daily smokers (Figure 1). Never smokers were defined as those adolescents who never tried a puff. We collapsed ex-smokers and experimental smokers and renamed them as current non-smokers due to lack information about quitting smoking in the survey. Current non-smokers were defined as smokers who tried at one puff or smoked previously but not within the past 30 days. Intermittent smokers were defined as adolescent smokers who reported smoking between 1 and 29 out of the past 30 days. Daily smokers were defined as those who reported smoking on a daily basis within the past 30 days. Based on the categories of smoking states, our outcomes were 1) ever smoking (current non-smokers, intermittent smokers, and daily smokers versus never smokers (reference)); 2) current smoking (intermittent smokers and daily smokers versus current non-smokers and never smokers (reference)); and 3) daily smoking (daily smokers versus intermittent smokers, current non-smokers, and never smokers (reference)), which were intended to examine associations of different predictors with crossing thresholds of the smoking states.

Individual-level variables included the adolescents’ demographic characteristics, parental SES, alcohol and substance use, and psychological status. We selected school grade instead of age which has missing data and treated it as a continuous variable. Self-rated academic achievement was categorized into five groups from very good (coding = 5) to very poor (coding = 1) and treated as a continuous variable. Based on the survey questionnaire, weekly allowance was coded per 10,000 KRW (about 9 USD, coding = 1) and ranged from 0 KRW to above 150,000 KRW (about 132 USD, coding = 16). Secondhand smoking at home was measured as a proxy for parental smoking by the number of days of exposure to cigarette smoke at home within the past seven days. Alcohol use also was measured by the number of days of drinking within the past seven days. Substance use was categorized in three groups: never, past use, and current use. Psychological status was assessed with two variables: stress status and depressive symptoms. Stress status was asked using a scale of usual stress status from “severely stressed” (coding = 5) to “never stressed” (coding = 1) and treated as a continuous variable. Depressive symptoms were assessed by asking whether the student experienced depressive symptoms within the year. A “yes” response was coded as 1, and “no” was coded as 0. Parental education and the Family Affluence Scale (FAS) were used to assess parental SES. Paternal and maternal education were categorized into three levels (middle school graduation or lower/high school graduation/college or higher). The FAS which was developed by the European Health Behaviour in School-aged Children (HBSC) Study [51] and is measured by four questions for adolescents: 1) having one’s own bedroom; 2) frequency of family trips per year; 3) the number of computers at home; and 4) the number of vehicles owned by one’s family. Despite differences between Korea and European countries regarding social, economic, and cultural backgrounds, the FAS was admitted as a valid and reliable measure of SES among Korean adolescents yielding compatible gradients in health with those by other SES indicators [52]. We categorized school type according to gender composition (boys-only, girls-only, and coeducation). For subgroup analysis among high school students, we also distinguished between general versus vocational schools.

### Statistical Analysis

A stratified two-level multilevel logistic regression model–individuals at level-1 nested with 800 middle and high schools at level-2–was fitted to estimate the contribution of contextual level characteristics on the adolescent smoking behaviors, taking into account the influence of individual-level determinants simultaneously. A random-intercept model was built separately for each outcome to estimate the associations of the individual- and the school-level factors with each adolescent smoking outcome. In model 1, we estimated the odds of current non-smoking, intermittent smoking, and daily smoking versus never smoking. In model 2, we estimated the odds of intermittent and daily smoking versus never and current non-smoking. Similarly, model 3 estimated the odds of daily smoking versus other smoking states. We used *MLwiN* (version 2.22) for the analysis. To obtain estimates and distributions of interest, we employed Markov Chain Monte Carlo (MCMC) function, a Bayesian approach implemented in *MLwiN*. The results are presented in odds ratios (OR) and 95% credible intervals (CI) with deviance information criterion (DIC) statistic as an indicator of model fit in a Bayesian framework. Models having smaller DIC are favored.

## Results

Table 1 shows distributions of variables across four smoking behaviors: never smokers, current non-smokers, intermittent smokers, and daily smokers. Male students outnumbered females in the every smoking category except never smokers. With each category of smoking intensity, average self-rated academic achievement tended to be lower, whereas weekly allowance, secondhand smoking at home, alcohol use, and stress tended to increase.

**Table 1:** Characteristics of never smokers, current non-smokers, intermittent smokers, and daily smokers among Korean adolescents.

		Total	Never smokers	Current non-smokers	Intermittent smokers	Daily smokers
		% (N)				
Gender	Male	51.0 (29,492)	45.8 (19,411)	59.7 (5,090)	67.6 (2,279)	75.6 (2,712)
	Female	49.0 (28,365)	54.2 (22,962)	40.3 (3,433)	32.4 (1,094)	24.4 (876)
Paternal education	< = Middle school	7.7 (4,460)	6.8 (2,900)	9.7 (827)	9.0 (305)	11.9 (428)
	= High school	44.6 (25,825)	43.0 (18,227)	48.0 (4,095)	49.0 (1,653)	51.6 (1,850)
	> = College	47.6 (27,572)	50.1 (21,246)	42.3 (3,601)	42.0 (1,415)	36.5 (1,310)
Maternal education	< = Middle school	7.7 (4,430)	6.9 (2,931)	9.2 (786)	8.8 (298)	11.6 (415)
	= High school	57.9 (33,513)	56.8 (24,072)	60.6 (5,166)	61.0 (2,057)	61.8 (2,218)
	> = College	34.4 (19,914)	36.3 (15,370)	30.2 (2,571)	30.2 (1,018)	26.6 (955)
Substance use	Never	99.6 (57,634)	99.8 (42,306)	99.5 (8,480)	98.8 (3,331)	98.0 (3,517)
	Past use	0.2 (139)	0.1 (43)	0.4 (32)	1.0 (34)	0.8 (30)
	Current use	0.1 (86)	0.1 (26)	0.1 (11)	0.2 (8)	1.1 (41)
Depressive symptoms	Yes	38.0 (21,982)	34.7 (14,686)	44.7 (3,813)	48.3 (1,629)	51.7 (1,854)
	No	62.0 (35,875)	65.3 (27,687)	55.3 (4,710)	51.7 (1,744)	48.3 (1,734)
		**Range**	**Mean (SD)**			
School grade		1–6	3.47 (1.70)	3.76 (1.61)	3.72 (1.54)	4.67 (1.23)
Self-rated academic achievement		1–5	3.20 (1.15)	2.85 (1.18)	2.64 (1.17)	2.34 (1.17)
Weekly allowance		1–16	2.72 (2.41)	3.26 (2.86)	3.81 (3.40)	4.86 (4.10)
Family Affluent Scale (FAS)		4–13	8.71 (1.78)	8.64 (1.80)	8.77 (1.82)	8.60 (1.83)
Secondhand smoking at home		0–7	1.31 (2.17)	1.75 (2.49)	2.00 (2.48)	2.65 (3.00)
Alcohol Use		0–7	0.23 (0.73)	0.61 (1.2)	1.47 (1.63)	2.52 (1.85)
Stress status		1–5	3.34 (0.93)	3.46 (0.96)	3.54 (0.96)	3.59 (1.00)

Table 2 shows the results of three two-level binomial logit models that estimated the odds of ever smoking, current smoking, and daily smoking. Male students were more likely to be ever smokers, current smokers, and daily smokers than females. Higher school grades (and thus increased age) were associated with an increasing risk of daily smoking and less so with increased risk of ever and current smoking. Adolescents who rated their academic achievement higher had lower risks of ever smoking, current smoking, and daily smoking. Of parental education variables, only paternal education of college or higher was significantly inversely associated with ever smoking. Higher FAS and more weekly allowance were associated with higher risks of smoking: the odds were similar across smoking behaviors. More exposure to secondhand smoking at home was associated with the higher risks of daily smoking, current smoking, and ever smoking. There were no significant interaction effects between the FAS and weekly allowance in predicting outcomes. Other individual-level factors including alcohol and substance use, stress status, and depressive symptoms were positively associated with risks of ever smoking, current smoking, and daily smoking.

**Table 2:** Odds ratios (OR) and 95% credible intervals (CI) based on two-level binomial logit models of ever smoking, current smoking, and daily smoking among Korean adolescents.

	Ever smoking			Current smoking			Daily smoking		
	Model 1 (2,3,4 vs. 1)§			Model 2 (3,4 vs. 1,2)§			Model 3 (4 vs. 1,2,3)§		
	OR	(95% CI)		OR	(95% CI)		OR	(95% CI)	
Gender (base: Female)									
Male	2.61*	(2.46	2.75)	3.15*	(2.90	3.42)	3.46*	(3.06	3.90)
School grade	1.07*	(1.05	1.09)	1.09*	(1.06	1.11)	1.37*	(1.31	1.42)
Self-rated academic achievement	0.73*	(0.71	0.74)	0.65*	(0.63	0.67)	0.59*	(0.57	0.62)
Weekly allowance	1.06*	(1.05	1.07)	1.07*	(1.06	1.08)	1.06*	(1.05	1.07)
Paternal education (base: < = Middle school)									
= High school	0.91	(0.84	1.01)	1.02	(0.89	1.16)	0.96	(0.83	1.14)
> = College	0.82*	(0.75	0.91)	0.94	(0.80	1.08)	0.85	(0.72	1.02)
Maternal education (base:< = Middle school)									
= High school	1.00	(0.92	1.08)	1.02	(0.90	1.14)	0.99	(0.85	1.17)
> = College	0.96	(0.87	1.05)	1.01	(0.89	1.15)	1.02	(0.86	1.24)
Family Affluent Scale (FAS)	1.03*	(1.02	1.04)	1.04*	(1.03	1.06)	1.04*	(1.01	1.06)
Secondhand smoking at home	1.08*	(1.07	1.08)	1.09*	(1.07	1.10)	1.12*	(1.10	1.13)
Alcohol use	1.77*	(1.73	1.81)	2.04*	(1.99	2.08)	1.94*	(1.89	1.99)
Substance use (base: Never)									
Past use	2.78*	(1.83	4.19)	2.51*	(1.67	3.86)	1.34	(0.78	2.20)
Current use	1.65	(0.94	2.93)	2.19*	(1.22	3.95)	4.36*	(2.47	8.09)
Stress status	1.10*	(1.08	1.14)	1.15*	(1.11	1.20)	1.14*	(1.10	1.20)
Depressive symptoms (base: No)									
Yes	1.41*	(1.35	1.48)	1.32*	(1.23	1.40)	1.26*	(1.15	1.38)
School type (base: Coeducation)									
Girls-only school	0.95	(0.86	1.04)	0.80*	(0.69	0.94)	0.69*	(0.56	0.87)
Boys-only school	0.90*	(0.81	0.99)	0.89	(0.78	1.02)	0.81*	(0.69	0.97)
Units: School	800			800			800		
Units: Individual	57857			57857			57857		
DIC	54461.21			29483.58			17038.41		

§ 1 = Never smokers; 2 = Current non-smokers; 3 = Intermittent smokers; 4 = Daily smokers.

DIC: deviance information criterion.

*p<.05.

Among contextual factors, attending single-sex schools was associated with lower risk of smoking compared with attendance of coeducation schools even after controlling for individual-level factors. Attending girls-only schools was associated with lower risks of daily smoking, current smoking, and ever smoking (marginally significant) than attending coeducation schools. Similarly, adolescents attending boys-only schools was associated with lower risks of daily smoking, current smoking (marginally significant), and ever smoking than attending coeducation schools. A subgroup analysis among high school students showed that students attending general high schools had lower risks of being ever smokers (OR = 0.50, 95% CI 0.44–0.55), current smokers (OR = 0.40; 95% CI 0.34–0.46), and daily smokers (OR = 0.39, 95% CI 0.32–0.47) compared to those attending vocational high schools even after controlling for individual-level factors.

## Discussion

This study explored the associations of individual- and school-level factors with different types of adolescent smoking using a multilevel analytical framework. We found that students attending coeducation schools or vocational high schools had higher risks of smoking for each type of smoking behavior compared to those attending single-sex schools or general high schools, respectively. We also showed that paternal and maternal education were not significant predictors of adolescent smoking, except for the inverse association between the highest level of paternal education (college or higher) and smoking. Higher FAS and weekly allowance were robustly associated with greater risks of all types of smoking behavior even after controlling for parental education and other confounders including stress status, depressive symptoms, and secondhand smoking at home.

Regarding school characteristics, this study reveals that students in coeducation schools were more likely to be ever smokers, current smokers, and daily smokers than those in single-sex schools. This may be explained by the unique pattern in development of peer networks among Korean adolescents. Korean adolescents generally lack opportunities to interact or socialize with opposite gender peers within the socially endorsed single-sex school system, which is an offshoot of the traditional Confucian culture that emphasizes students’ academic success. Under such an educational environment, adolescent peer networks tend to be restricted within their classes or schools [53]. Students spend so much time studying in schools or private academic institutes [54], [55] that they barely have time left to spare for leisure or social activities [53], [55]. Moreover, most parents frown upon their children making opposite sex friends because of concerns about distraction from studying or concerns about sexual morality [56]. Consequently, Korean students in single-sex schools tend to have more same-sex friends than those in coeducational schools [56]. On the other hand, given that adolescents in coeducation schools have more opportunities to interact with those of the opposite sex, adolescents in these schools may use cigarettes to project an image of maturity and sophistication in the presence of their peers. It is well established that adolescents tend to regard smoking as a symbol of maturity or attractiveness [57], which is reinforced by advertising, and potentiated in the presence of the opposite sex [58], [59].

From the subgroup analysis, we found that students attending vocational high schools had higher risks of being ever smokers, current smokers, and daily smokers than those attending general high schools. Given that Korean society is a highly academically stratified society, the uneven smoking risks between these two school types may be explained by discrimination due to difference in academic achievement [60], [61]. Students in vocational high schools may experience or perceive discrimination due to prevalent attitudes that favor higher educational attainment in Korean society [60], [61]. Korean students have to choose either general or vocational high schools when they graduate from middle schools, depending mainly on their academic scores rather than their aptitudes or interests [62]. Given that more than 70% of Korean high school students enter universities in pursuit of higher educational attainment, attending vocational high schools tends to be equated with lower academic achievement. In the highly academically stratified society, students who graduate from vocational high schools also experience discrimination, including lower employment opportunities, significantly lower salary, and longer years for promotion compared to those who graduated general high school or have an undergraduate degree [60], [61]. To cope with uneasiness and stress from discrimination, students attending vocational high schools may individually use cigarettes [63], [64]. At school-level, these students may also socialize with each other, sharing a norm that reinforces or endorses smoking to lessen the stress from discrimination, as well as to signal their solidarity with each other [65], [66].

We also found several individual-level factors affecting adolescent smoking. After controlling for a comprehensive range of possible confounders such as stress [7], depression [33], weekly allowance [34], and exposure to secondhand smoking as a proxy for parental smoking [35], parental education was not a significant factor for adolescent smoking except paternal education of college or higher. By contrast, higher FAS was robustly associated with higher risks of ever smoking, current smoking, and daily smoking. These findings are inconsistent with several previous studies [16], [67], [68] and may suggest another pathway through which parental SES may be linked with adolescent smoking. High FAS may make adolescents less susceptible to conventional values, and accordingly their increased risk-taking may promote smoking [69]. Previous studies on adolescence reported that perceptions of socioeconomic affluence may lead adolescents to feel relatively free to deviate from social norms [70] and to recognize themselves as exceptional or above social norms [70], [71]. In other words, adolescents may initiate and increase smoking based on their feelings of prestige, or because they believe themselves to be immune from punishment or societal disapproval [70], [71].

We found that more weekly allowance was robustly associated with increased risks for ever smoking, current smoking, and daily smoking even after controlling for confounders including psychological factors and parental SES factors. Our finding is mostly consistent with West et al. [32] indicating that higher disposable income is a risk factor for adolescent smoking. Moreover, the association between higher allowance and smoking did not vary by parental SES background. This finding suggests several likely explanations. Adolescents with more allowance may have more ability to buy cigarettes or may be more able to participate in activities facilitating smoking such as using internet café, pub, or Karaoke [72]. Higher allowances may also be another trigger for the perception of prestige as we mentioned above [30]. This result may point out an important policy implication to restrict the access of Korean adolescents to tobacco products: increasing tobacco taxation for effective tobacco control. The tobacco prices in Korea (about 2.2 USD) were ranked as the 7th lowest among 32 developed countries (mean = 4.42 USD) [73]. The price of cigarettes has risen only 45% during the 14 years since 1994 [74] due to the delayed implementation of tobacco tax increasing. The low tobacco price might have seduced adolescents of high allowance to smoke more. Future research is needed to better understand the relationship between allowance, parental SES, and adolescent smoking within the broader context of anti-smoking policies such as taxation and restriction on youth access to tobacco products.

Our findings of other factors associated with adolescent smoking were consistent with past studies. Higher grade [75]–[77] and lower self-rated academic achievement [44], frequent alcohol [44], [72] and substance use [6], higher level of stress [78]–[80], and more depressive symptoms [81], [82] were each associated with ever smoking, current smoking, and daily smoking.

Lastly, we examined whether different factors were associated with specific smoking behaviors. However, our results mostly do not support the hypothesis of differential predictors: the different smoking behaviors (with the exception of paternal education) share similar risk and protective factors.

Several limitations should be noted when interpreting our findings. Firstly, our study is cross-sectional and we cannot infer causality. Secondly and related to the previous, our cross-sectional study also cannot exclude selection effects, i.e. the association between vocational schools and higher rates of smoking may not be a reflection of the school environment influencing the risk of smoking. It may be that students who begin smoking at a younger age are less interested in an academic career and select into vocational schools [83], [84]. Nevertheless, this does not detract from the need to focus on vocational schools as the locus of anti-smoking efforts. Thirdly, students may have answered in a socially desirable manner (e.g., reporting lower smoking rates or hiding the initiation of smoking) despite the assurance of anonymity in the responses. Fourthly, we were able to use just two variables on school characteristics because other related questions were not asked in the survey. Future investigation is needed to identify additional school characteristics associated with adolescent smoking especially in Asian contexts. Lastly, there may be unknown confounders we did not control for in analysis.

In summary, being male, in higher grades, having lower self-rated academic achievement, having more weekly allowance, higher FAS, more frequent exposure to secondhand smoking at home, more frequent alcohol and substance use, higher stress status, and experiencing depressive symptoms were each individual-level risk factors of ever smoking, current smoking, and daily smoking. Although some associations were marginally significant, we also found that attending coeducation schools and vocational high schools were associated with higher smoking risk. Advocates for tobacco control should consider these factors at school as well as individual level to develop more effective policies and interventions.
